# Spatial and temporal pattern of wildfires in California from 2000 to 2019

**DOI:** 10.1038/s41598-021-88131-9

**Published:** 2021-04-22

**Authors:** Shu Li, Tirtha Banerjee

**Affiliations:** Department of Civil and Environmental Engineering, University of California, Irvine, CA 92697 USA

**Keywords:** Natural hazards, Fire ecology

## Abstract

The environmental pollution, property losses and casualties caused by wildfires in California are getting worse by the year. To minimize the interference of wildfires on economic and social development, and formulate targeted mitigation strategies, it is imperative to understand the scale and extent of previous wildfire occurrences. In this study, we first investigated the temporal distributions of past wildfires in California divided by size and causes and analyzed the changes observed in the past two decades against the last century. The trend of wildfires in different time scales (yearly and monthly), as well as the distribution of wildfires across different spatial scales (administrative units, climate divisions in California from 2000 to 2019) were also studied. Furthermore, to extract the significant variables on the risk of wildfire occurrence, multivariate analyses of environmental and human-related variables with wildfire densities were carried out. The results show that the wildfire density distribution of the burned area in California conforms to the characteristics of the Pareto distribution. Over the past two decades, the frequency of small (< 500 acres), human-caused wildfires has increased most rapidly, and they are widely distributed in central and western California. The wildfire season has lengthened and the peak months have been advanced from August to July. In terms of the variables related to the risk of wildfire occurrence, the temperature, vapor pressure deficit, grass cover, and the distance to roads are crucial. This study reveals the relationship between environmental and social background conditions and the spatial-temporal distribution of wildfires, which can provide a reference for wildfire management, the formulation of future targeted wildfire emergency plans, and the planning of future land use in California.

## Introduction

As one of the most frequent natural disasters in California, wildfires have caused great damage to the environment, economy and society in recent years^1,2^. Especially in the past two decades, changes in climate and land utilization caused by human activities have not only extended the wildfire season, but also significantly increased the severity and burned areas of wildland fires^3^. At the same time, the expansion of the wildland-urban interface (WUI) areas caused by rapid social development and sustained population growth has greatly increased the number of residents and buildings affected by wildfires, which has further aggravated the damage imparted to the human society from wildfires^4,5^. According to the data from the wildfire Redbooks published by the California Department of Forestry and Fire Protection (CAL FIRE), despite significant administrative investments in wildfire suppression and management in recent years, the property loss caused by wildfires has not been significantly reduced in California^6^.

The development and implementation of proactive fire prevention policies can effectively reduce the probability of wildfire ignition, the risk of extreme fires, and the social and economic losses caused by wildfires. The formulation of effective policies entails a full understanding of the spatial and temporal distribution of different types of wildfires (natural and human-caused), the differences in their impact on human communities, and their various influential factors. To this end, the dominant causes and drivers of California wildfires in different periods and regions have already been analyzed by several researchers. Faivre et al.^7^ used the logistic and Poisson regression models to analyze fires in Southern California National Forests from 1980 to 2009. The results indicated that the distance from wildfire ignition points to houses and highways, and the terrain slope were the leading factors that explain ignition frequency. Nevertheless, the study was limited to Southern California and focused only on the spatial distribution of wildfires.

Keeley and Syphard’s analysis^8^ of the spatial distribution of wildfires over the past 100 years in California found that the frequency of wildfires declined greatly after 1980, but there has been no corresponding significant change in the total annual burned area. Prior to 1980, the main cause of wildfires in most parts of California was human activity. However, in recent decades, most man-made ignition sources other than power lines have become less frequent, and the positive correlation between wildfire frequency and population distribution has been less pronounced in recent years than it was in the last century. Therefore, the relative importance of relevant variables in influencing wildfire occurrence varies over time. However, this study only focused on the spatial distribution of wildfires with different causes, and did not analyze other factors affecting the spatial distribution of wildfires in detail.

Williams et al.^9^ demonstrated that from 1972 to 2018, the drying of forest fuels due to human-induced climate warming has greatly increased the area of California’s forest-fires, especially in the summer months. Thus, wildfire management not only needs to reduce and prevent direct anthropogenic fire sources, but also needs to deal with changes in environmental risks such as human-induced climate change. Effective fire management therefore requires a comprehensive and near-real-time analysis of fire risks in the local natural environment, the scope and intensity of human activities, and the distribution of combustible fuels^10^.

Nevertheless, most of the current literature has been found to be focused on the historical distribution of wildfires, with the study periods ranging from 1910 to 2019. However, in the last two decades, the climate and the distribution of human communities in CA have changed greatly, which should have a significant impact on the ignition, spread and distribution of wildfires. The behavior and patterns of wildfires in California over the past two decades have not been adequately explored. Also, the current studies lack a detailed analysis of wildfires across California and their seasonality. From the perspective of wildfire management, the statistical analysis procedures, classification techniques, and analyses criteria are not consistent among different fire management agencies, administrative units, and relevant government departments, which makes it difficult to coordinate firefighting and prevention. Moreover, due to the complexity of the anthropogenic ignition causes, human-caused wildfires need to be further classified to formulate more targeted policies.

Considering the casualties, economic losses and environmental pollution caused by the combustion and spread of wildfires, it can be more cost-effective to pay more attention to preventing human-caused wildfires than putting them out^11^ (it is also worth noting that managed prescribed fires and low-intensity natural wildfires are actually beneficial from an ecological perspective for particular landscapes). To develop proactive wildfire prevention measures, it is necessary to conduct a detailed analysis of the current spatiotemporal distribution of wildfires with different causes, especially for large wildfires. Furthermore, preventing human-caused wildfires at source requires more detailed classifications of how, where and why these wildfires start, and identifying the social factors behind them^12^. Aiming at this gap, the research scope was expanded to the entire State of California in this study, and CAL FIRE’s multi-agency integrated wildfire records were selected as the original data to conduct a unified temporal and spatial distribution analysis. For the sake of eliminating the inconvenience caused by the differences in the classification of wildfires between various agencies, the administrative units covering the whole of California and the wildfire causes classification records provided by CAL FIRE were used as the basis of spatial analysis.

The aforementioned publications have established close relationships among some environmental and social factors and the probability of wildfires occurrences, which are critical in the formulation of wildfire prevention and management policies. However, the contribution of these external factors to the risk of wildfires is not entirely consistent across time, region, and cause of wildfires. In order to achieve a better understanding of California’s current wildfire situation, it is necessary to investigate the distribution of different causes of wildfires in the past two decades in detail. The importance of various external factors in explaining California’s wildfire occurrences needs to be analyzed as well.

In light of this context, this study mainly answers the following questions: (1) Has the probability density distribution of wildfire size changed over the past century? (2) what is the temporal distribution trend of wildfire frequency and burned area between different fire sizes and causes within the last two decades; compared to the earlier 80 years, that is from 1920 to 2000, what has changed? (3) What are the spatial distribution characteristics of wildfire density with different causes in 2000–2019 and 1920–1999? (4) The correlation and importance of the explanatory natural and social variables with the risk of wildfire occurrence.

## Results and discussions

California has a vast area and spans ten latitudes, and its internal geographical conditions and climate conditions vary widely^13^. Therefore, the California wildfires in history differed greatly in their frequency, size, intensity and extent of damage^8^. As the California wildfires are growing fiercer, they have become a hot topic worldwide. However, there is still a long way to go before the general conclusions from the wildfire literature can be applied in practice. For example, how the analyses of which types of wildfires are increasing the fastest can be used to guide the amendment of wildfire management policies? and how to guide fire fighting methods based on the results of the wildfire dominant factor model? To provide some practical reference for wildfire management work, we grouped the wildfires according to size (large fires, small fires) and ignition cause (natural fires and human-caused fires), and discussed their distribution characteristics separately using the administrative units from CAL FIRE and the weather division of California from the National Oceanic and Atmospheric Administration (NOAA) as the base map. While focusing on wildfires in the past two decades, the distribution of wildfires from 1920 to 1999 was used as prior information for comparison.

### Wildfire size distribution

**Figure 1:**
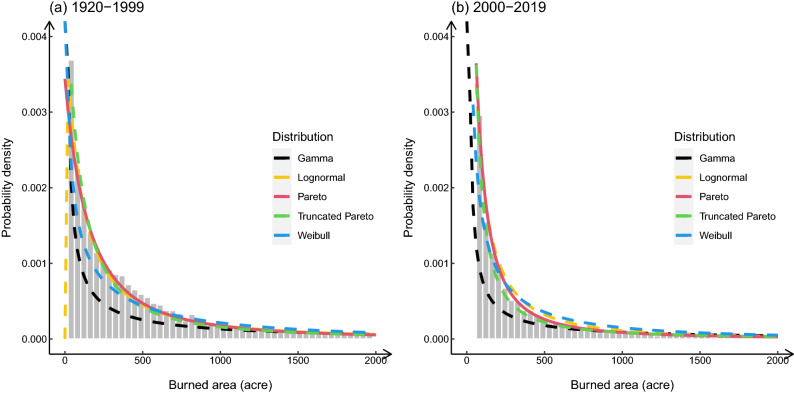
The empirical histogram of wildfire size and the typical heavy tailed distribution fitting curves for wildfires in (**a**) 1920–1999 and (**b**) 2000–2019. The wildfire sizes are in acres (1000 acre = 4.05 km$^2$). The curve with different colors represent different types of distribution, the black, yellow, red, green, and blue curves represent the fitting result of Gamma, Log-normal, Pareto, Truncated Pareto, and Weibull distribution, separately. The tail of the distribution was truncated from the burned area of 2000 acres to show the fitting difference between different distributions.

The burned area of wildfires is an important indicator of their destructive power. Several studies have shown that 1% of large and extreme wildfires are responsible for 90% of the total damage caused by wildfires^14,15^. Besides, the Probability density distribution of wildfire burned area, that is the wildfire size, has an obvious heavy tail feature. Research from Strauss et al.^16^ and Holmes et al.^17^ indicate that the wildfire size distribution fits the Pareto distribution well. Based on their conclusions, five common heavy-tailed distributions were selected (which are Gamma, Lognormal, Pareto, Truncated Pareto and Weibull distribution) to fit the wildfire size distribution throughout California within the eighty years before year 2000 and twenty years after year 2000s, seeking the best description of the California wildfire size distribution. The estimated parameters and the goodness of fit test results are shown in Tables 1 and 2. The empirical wildfire size distribution and the fitting curve are shown in Figure. 1. Fig. 1 shows that the wildfire size distribution did not change much from the last century to the present. Also, all these fitting curves can capture the main feature of the empirical distribution. Table 1 lists the estimated shape and scale parameters for each distribution. It can be found that the shape parameter of current wildfire size distribution ($\alpha$) decrease compared to the historical wildfires. The value of shape determines the thickness of the tail. A smaller shape value means a thicker tail. In the context of wildfires, it means the probability density of large wildfires increase. Table 2 shows the goodness of fit for each distribution by Akaike Information Criterion (AIC), Kolmogorov-Smirnov (K-S) and Cramer-VonMises (CvM) test score. For all the tests, the smaller the value of the test score, the better the fit. Among these five fits, the lognormal distribution is the best for wildfire size description in 1920–1999, following by the Pareto distribution; while the best fitting distribution in 2000–2019 changes to the truncated Pareto, the second-best fitting result is still from the Pareto distribution. Therefore, Pareto is appropriate to summarize the general feature of wildfire size distribution in California.

**Table 1:** Heavy-tailed distribution fitting results of wildfire size distribution.

Distribution	Probability density function	Wildfires in 1920–1999		Wildfires in 2000–2019	
		Shape($\hat{\alpha }$)	Scale($\hat{\beta }$)	Shape($\hat{\alpha }$)	Scale($\hat{\beta }$)
Gamma	$\frac{\beta ^{\alpha }}{\Gamma (\alpha )}x^{\alpha -1}e^{-\beta x}$	0.2085	0.0002	0.1251	0.0002
Lognormal	$\frac{1}{\sqrt{2\pi }\beta x}exp[-\frac{1}{2\beta ^2}(lnx-\alpha )^2]$	5.6400	1.6800	4.7900	1.8300
Pareto	$\frac{\alpha \beta ^\alpha }{x^{\alpha +1}}$	1.0800	313.2500	0.7280	61.9350
Truncated pareto	$\frac{\alpha \beta ^\alpha x^{-\alpha -1}}{1-(\frac{\beta }{H})^\alpha }$	0.6960	149.3510	0.4820	52.6270
Weibull	$\frac{\alpha }{\beta }(\frac{x}{\beta })^{\alpha -1}e^{-(x/\beta )^\alpha }$	0.6050	658.5420	0.4970	319.2410

**Table 2:** Goodness-of-fit test results of Akaike Information Criterion (AIC), Kolmogorov–Smirnov (K–S) test, and Cramer-Von Mises (CvM) test for heavy-tailed distribution fitting.

Distribution	Wildfires in 1920–1999			Wildfires in 2000–2019		
	AIC	K-S	CvM	AIC	K-S	CvM
Gamma	186,942.2	0.2967	277.72	75,814.81	0.4713	240.94
Lognormal	178,499.6	0.0234	1.37	69,481.42	0.0869	13.24
Pareto	178,987.2	0.0333	2.38	69,470.16	0.1033	5.69
Truncated pareto	177,643.5	0.0317	3.40	67,562.65	0.0129	0.15
Weibull	180,347.5	0.0764	19.02	71,096.81	0.1637	31.55

**Figure 2:**
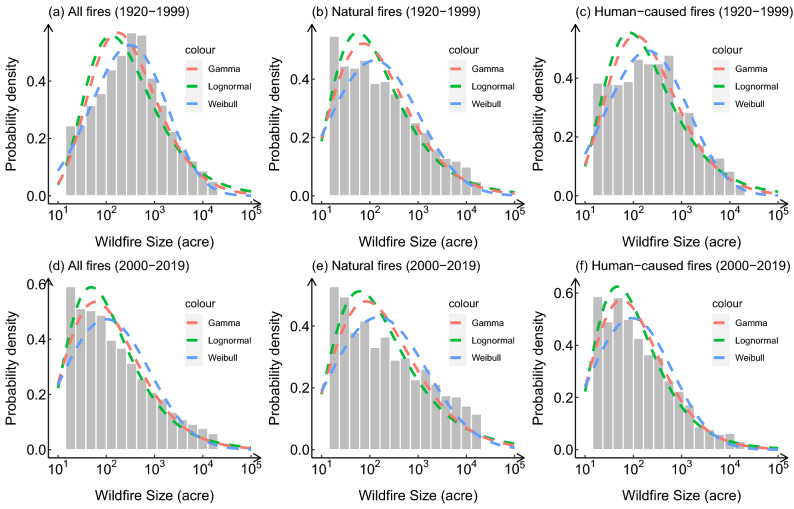
Logarithm of California wildfire size empirical distribution in 1920–1999 and 2000–2019. The Gamma, Lognormal and Weibull distribution fitting results are indicated by the red, green and blue dash lines. The wildfire sizes are in acres (1000 acre = 4.05 km$^2$). (**a**–**c**) are the historical wildfires from 1920 to 1999, (**d**–**f**) are the wildfires from 2000 to 2019; (**a**,**d**) are the distribution of all wildfires, (**b**,**e**) are the distribution of natural wildfires, (**c**,**f**) are the distribution of human-caused wildfires.

To further explore the variation of wildfire size distribution within the entire state of California, the probability density of the logarithm of wildfire size was plotted for 1920 to 1999 and 2000 to 2019. As shown in Fig. 2, wildfires in 1920–1999 were mostly about 100–1000 acre (0.40–4.05 km$^2$) in size; while during 2000–2019, the number of small fires increased significantly,

the majority of wildfire sizes were in the range of 10–100 acres (0.04–0.40 km$^2$). Wildfires were also divided into natural wildfires and human-caused wildfires based on their ignition causes. The red, green and blue dashed lines in the figure delineate the fitting results of Gamma, Lognormal and Weibull distribution separately, which capture the distribution characteristics for each type of the wildfires. The fitting parameters and the goodness of test results were attached in the supplementary information (Table S1). Figure 2b,e show that although the overall shape of the distribution of natural wildfires in 1920–1999 and 2000–2019 are similar, the proportion of extreme wildfires larger than 10,000 acres (40.47 km$^2$) has increased significantly in the last two decades. From Fig. 2c,f, it can be found that the shape of the fire size distribution of human-caused wildfires differs greatly, which is the result of the rapid increase of the proportion of small fires. Although human activity directly or indirectly ignited 44$\%$ of wildfires in the United States^18^ and 39$\%$ of wildfires in California (as shown in the statistical summary in Table 3), they are generally easily contained in the initial attack^19^. The rapidly growing population in California has led to increased human activities and community coverage, which has increased the incidence of human-caused wildfires^20^. However, the expansion of human land has reduced the continuity, which is essential for the spread of wildfires^21^. Also, the improvement of wildfire monitoring and fire fighting ability has made most of the small human-caused wildfires able to be extinguished during the first 24 h after discovery^19^. Together, these reasons lead to the rapid increase in the frequency of small human-caused fires in the past two decades.

**Table 3:** Statistical summary of wildfire ignition causes in CA from 2000 to 2019.

Causes	Lightning	Human-caused			Miscellaneous	Unknown
		Transportation	Human activity	Construction		
Number of wildfires	1,530	419	1,754	302	746	1,585
Percentage	24.15	6.61	27.68	4.77	11.77	25.02

**Figure 3:**
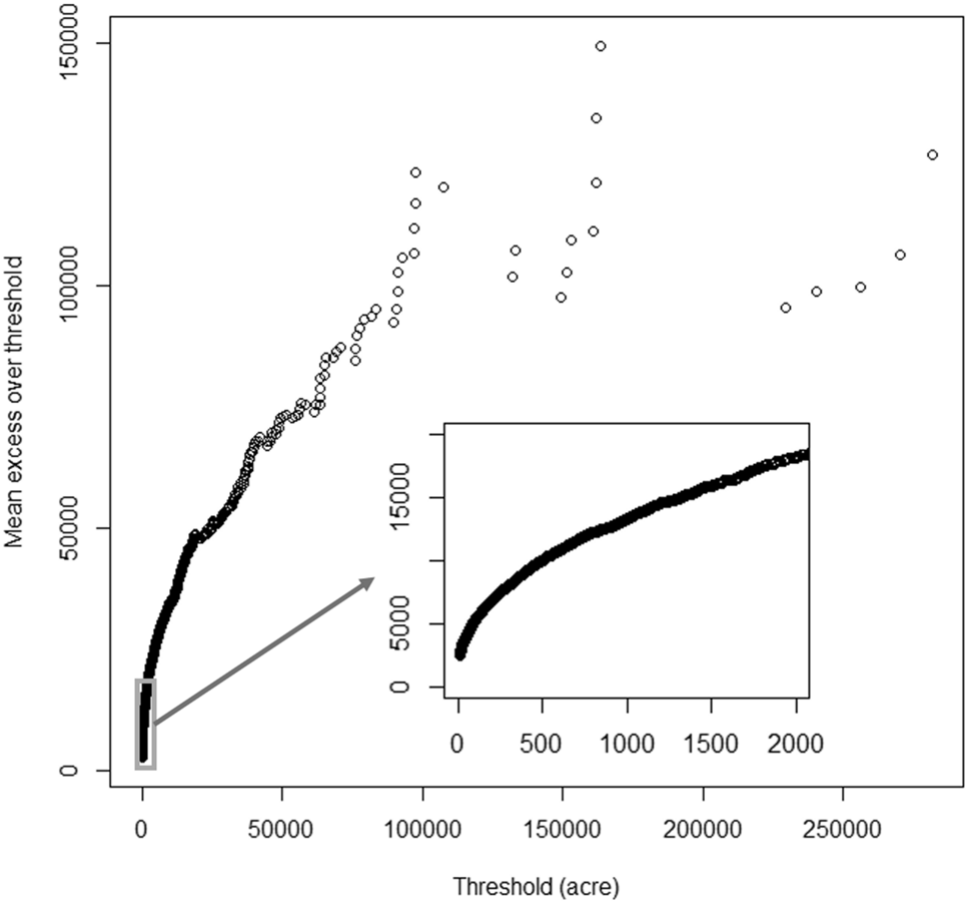
Mean excess plot for wildfires burned areas.

Large and small fires are not only very different in the probability density distribution characteristics but also in prevention measures, response methods, and resources needed to be invested in fire fighting^22,23^. In order to discuss the spatiotemporal distribution of large and small wildfires, it is critical to determine the threshold of large wildfires. Therefore, the mean excess plot shown in Fig. 3 was used to determine the threshold of the large fire. The linear part’s starting point is the threshold of the extreme value in the original distribution^17,24^. As shown in Fig. 3, 500 acres (2.02 km$^2$) would be appropriate to separate the large fires and small fires for the entire California. Also, as shown in Fig. 1, 500 acres is an appropriate starting point of the heavy tail. Based on the historical record from CAL FIRE, the frequency of large wildfires accounted for 19.68 $\%$ of the total (1247 out of 6336 wildfires), while the burned area of large wildfires accounted for 97.04 $\%$ of the total burned area (13,089.68 out of 13,488.19 thousand acres, that is 52,972.05 out of 54,584.77 km$^2$) in the past two decades. According to the size class of fire defined by national wildfire coordinating group (NWCG), the large fire in this study refers to the wildfires of or larger than class E.

### Temporal variation of wildfires in CA from 1920–1999 and 2000 to 2019

**Figure 4:**
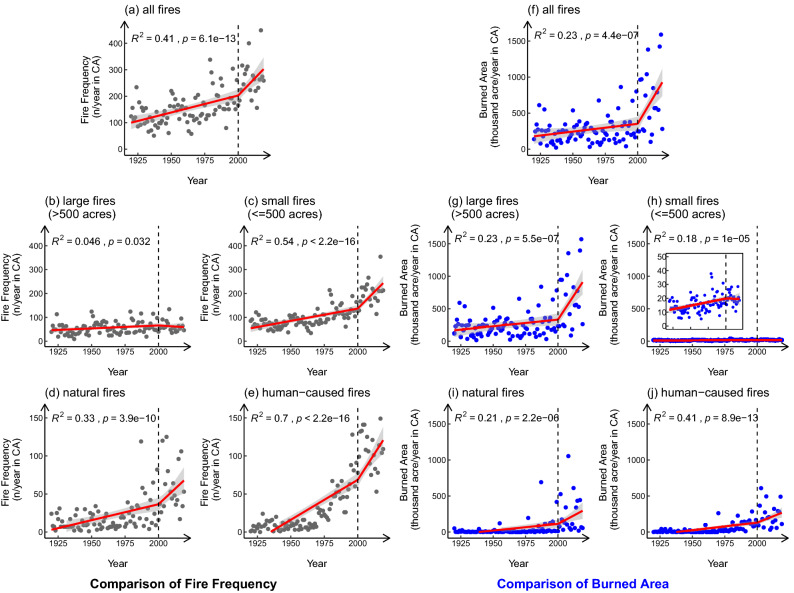
Temporal distribution of wildfire frequency and burned area from 1920 to 2019. The red line indicates the segmented linear regression results for 1920–1999 and 2000–2019. The gray areas indicate the 95$\%$ confidence interval. $R^2$ represents the coefficient of determination and p represents the p-value. (**a**–**e**) are the temporal distribution of wildfire frequency, (**f**–**j**) are the temporal distribution of the burned area of wildfires; (**a**,**f**) are the distribution for all wildfires; (**b**,**g**) are plots of large fires, which have the burned area larger than 500 acres (2.02 km$^2$), while (**c**,**h**) are plots of small fires, which have the burned area in the range of 10 acres (0.04 km$^2$) to 500 acres (2.02 km$^2$); (**d**,**i**,**e**,**j**) divided wildfires into natural fires and human-caused fires. The small plot in (**h**) zooms in to the burned area of 0–50 thousand acres.

Based on the wildfire history records provided by the CAL FIRE Fire Perimeter database, the frequency and burned area of wildfires in CA from 1920 to 2019 were extracted, and separated into two time periods: 1920–1999 and 2000–2019. California has seen an average of 317 wildfires a year over the past 20 years, which were included in the Fire Perimeter database, burning an average of 674,410 acres (2,729.24 km$^2$). Figure 4 shows the changes in the annual wildfire frequency (a–e) and burned area (f–j) over time. The red lines represent the segmented linear regression trend in 1920–1999 and 2000–2019, separately. The grey areas depicted the 95$\%$ confidence interval. Comparing the slope of the fitting line, it is apparent that in most cases, the frequency and burned area growth of wildfires in the past two decades are much higher than that during the 80 years in history, if the breakpoint is fixed to the year 2000. Also, the 95$\%$ confidence intervals of the regression lines over the past two decades are generally larger than that between 1920 and 1999. Although the sample size in these two time periods is different, it can be seen from the spread of data points that the uncertainty of wildfire frequency and burned area have increased significantly in the past two decades. From the view of fire frequency, the rapid increase in the number of small fires brings greater uncertainty than that of large fires, and the uncertainty of natural fires is higher than that of human-caused fires. In terms of the burned area, the uncertainty comes mainly from large wildfires and natural wildfires. When it comes to the increase rate, Fig. 4b,c,g,h show that in the large and small wildfire group, the accelerated increase of wildfire frequency was mainly contributed by the small fires, while the accelerated increase of burned area was from the large fires. The frequency of large wildfires and the burned area of small wildfires in the recent 20 years even have the trend of decrease. This trend suggests that it would be efficient for the fire management department to pay more attention to the regions with the potential risk of extreme fires and prevent small fires from burning continuously and becoming large fires. Figure 4d,e,i,j display the trend for the natural and human-caused wildfires. The increase of the human-caused wildfire frequency is much faster than that of the natural wildfires in both time periods. However, the increases in the burned area due to the increasing frequency of wildfires with different causes are similar. It shows that the human-caused small wildfires have the strongest growth trend in the recent twenty years. In the view of wildfire management, while human activities increase the likelihood of wildfires ignition, large natural fires are more threatening in terms of size and destruction.

California’s Mediterranean climate is characterized by hot and dry summers, which leads to a high wildfire ignition risk^25,26^. Also, the hot and dry Santa Ana wind events have accelerated the spread of wildfires each fall^27^. The precipitation in California was concentrated in the winter, and the temperature was moderate^28^, allowing wildland vegetation to grow fast and storing fuel for next year. However, the significant climate change after the year 2000 has affected the seasonal distribution of wildfires.

**Figure 5:**
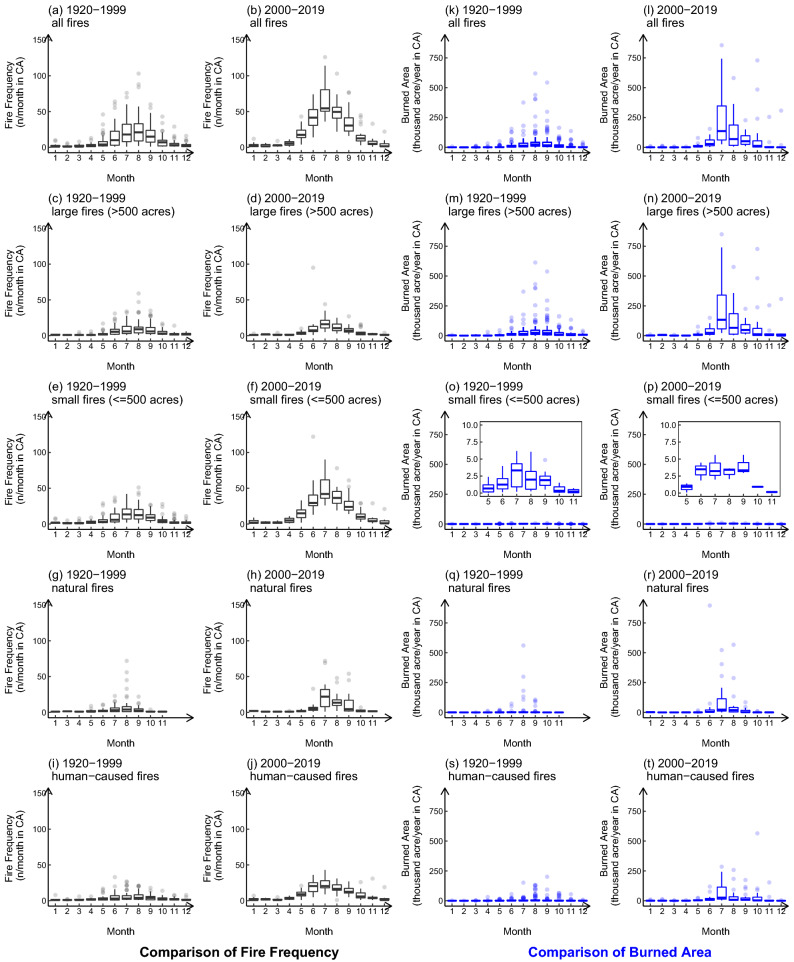
Seasonal variation of wildfire frequency and burned area from 1920 to 2019. The threshold of large and small wildfires is 500 acre (2.02 km$^2$). (**a**–**j** show the seasonal variation of fire frequency, (**k**–**t**) show the seasonal variation of burned area; (**a**,**b**,**k**,**l**) are plots for all CA wildfires, (**c**–**f**) and (**m**–**p**) divided fires into large and small fire size group, (**g**–**j**) and (**q**–**t**) divided fires into natural and human-caused wildfire groups. The small plots in (**o**) and (**p**) zoom in to the burned area of 0–10 thousand acres.

Figure 5 compiles box plots of the seasonal variation of wildfire frequency and burned area distribution in 1920–1999 and 2000–2019, which were divided into different groups by size and ignition cause as well. The boxes and points in the plots represent the wildfire frequency or total burned area in this month each year. In general, the peak season for wildfires was late summer and early autumn. In terms of the frequency, from 1920 to 1999, the wildfire season started in June, and the most frequent occurrence was observed in August. In most years, the number of wildfires in July and August were similar, followed by June and September. However, from 2000 to 2019, the frequency of wildfires in July increased significantly and became much more considerable than in other months. Meanwhile, the start of the wildfire season has also advanced to May, and the duration has extended. From May to September, the overall fire frequency of all wildfires, large wildfires, and small wildfires increased each month. The number of natural fires also increased between June to September. The frequency of human-caused wildfires, on the other hand, increased each month. Similar to the previous discussions, the increase of wildfire frequency in July in the past two decades mainly came from small fires and human-caused wildfires. It is worth noting that there has been a major increase in the natural wildfires in July in the past two decades. In terms of the burned area, the month with the largest total burned area of wildfires in 2000–2019 has been advanced to July, compared to August in 1920–1999. Natural wildfires and human-caused wildfires contributed similarly to the burned area growth. There is no noticeable change in the total burned area in months other than the wildfire season.

### Spatial distribution of wildfires in CA from 2000 to 2019

**Figure 6:**
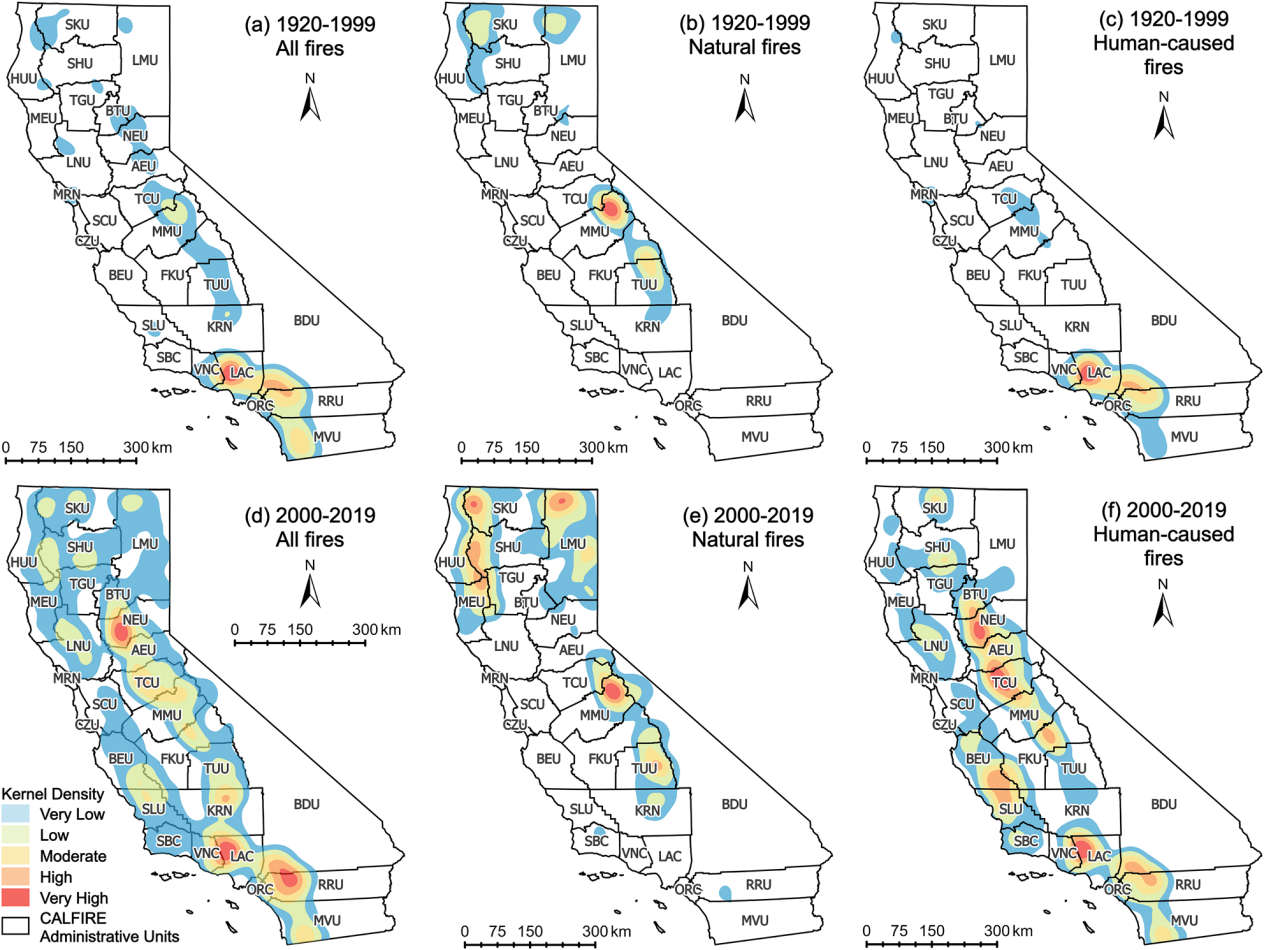
Kernel density distribution of wildfire occurrence in CA during 1920–1999 (**a**–**c**), and 2000–2019 (**d**–**f**). (**a**–**f**) are wildfire density distribution maps for all wildfires, natural wildfires and human-caused wildfires in CA, separately.

**Figure 7:**
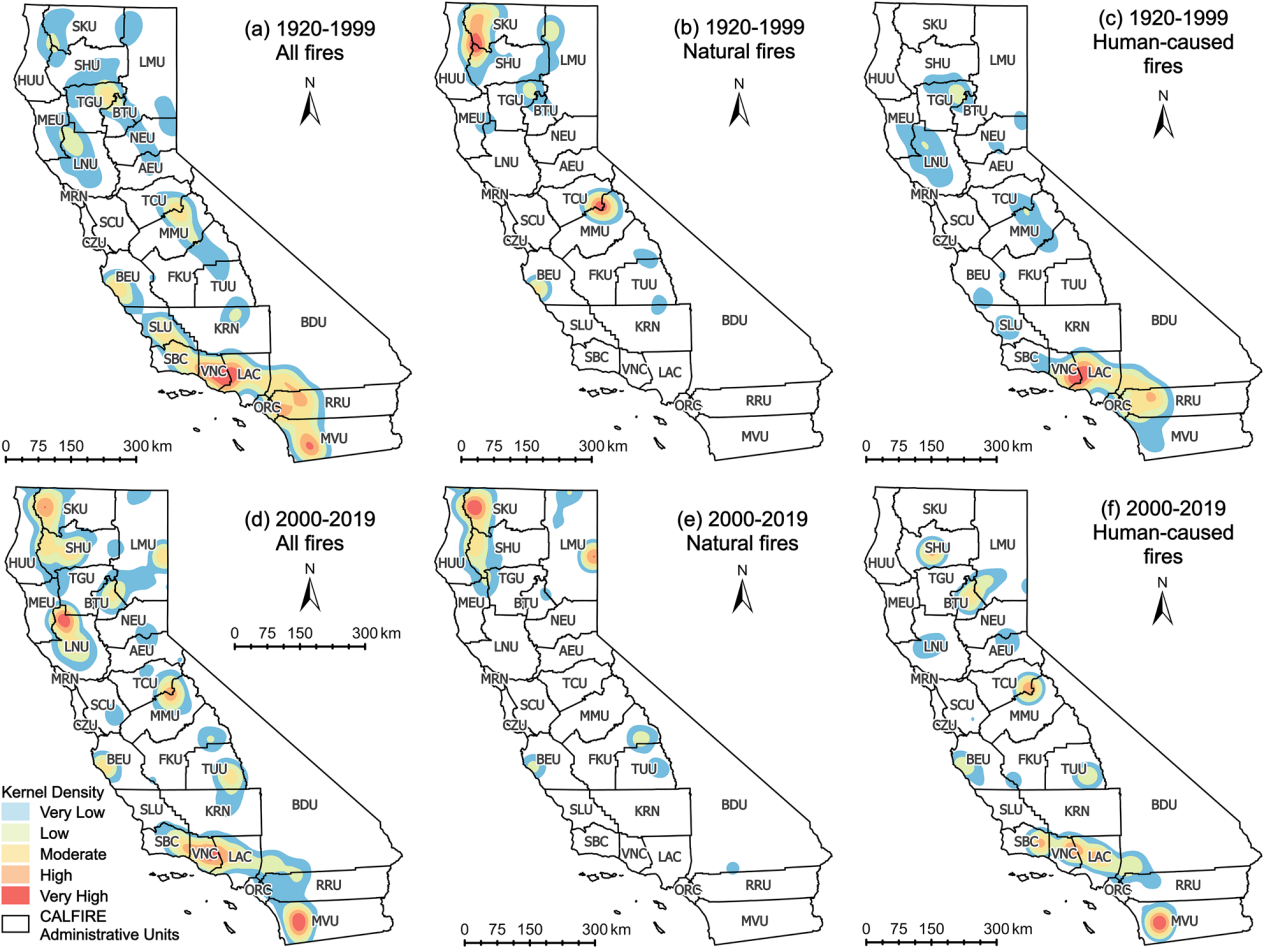
Kernel density distribution of burned area weighted wildfire occurrence in CA during 1920–1999 (**a**–**c**), and 2000–2019 (**d**–**f**). (**a**–**f**) are wildfire density distribution maps for all wildfires, natural wildfires and human-caused wildfires in CA, separately.

CAL FIRE has 21 operational units throughout the state that are designated to address fire suppression over a certain geographic area and six ‘Contract Counties’ (Kern, Los Angeles, Marin, Orange, Santa Barbara and Ventura) for fire protection services. Due to the complex environmental and terrain conditions in California, the risk of wildfires varies significantly from region to region, and the causes of extreme wildfires are also completely different. In order to provide fire managers with more effective fire suppression measures, this study used kernel density estimation (KDE) to analyze hot spot regions of all the wildfires, natural fires and human-caused fires from 2000 to 2019, the KDE for wildfires in 1920–1999 were also added for comparison. The resolution of KDE analyses was 500 m. The results are shown in Figs. 6 and 7. Figure 6 treated all the fires equally, and shows the spatial density of wildfire numbers; while Fig. 7 weighted the wildfires with their burned area, and represents the burned area-weighted spatial density of wildfire occurrence.

Comparing the spatial density distribution of all wildfires in different time periods in this study, as shown in Fig. 6a,d, it is evident that the coverage of wildfire occurrence has increased significantly. From 1920 to 1999, the only hot spot with a very high wildfire density was Los Angeles County (LAC). In the past two decades, not only did the hot spot of LAC expand to Ventura county (VNC) but also the wildfire density in the southwest corner of Riverside Unit (RRU) and San Diego Unit (MVU) on the south coast and the southwest corner of San Bernardino Unit (BDU) have grown to a very high level. In the eastern part of the San Joaquin Drainage under the central California climate division, namely the Sierra Nevada Mountains (identified in Fig. 10), wildfire density has increased from very low to very high. Among them, Nevada-Yuba-Placer Unit (NEU) and Tuolumne-Calaveras Unit (TCU) are the newly emerged high-density wildfire regions. Moreover, the spatial density distributions were grouped by causes, and Fig. 6b,e represent the natural wildfires, and c,f represent the human-caused wildfires. It can be found that while the high-density areas of natural wildfires have not shifted in both time periods, the density has increased. In contrast, the density of human-caused wildfires has increased notably in western and central California in the past two decades. Before the year 2000, there were almost no human-caused wildfires along the west coastline, but almost every county along the west-coast is characterized by an increase of human-caused wildfires in the past two decades. San Benito-Monterey Unit (BEU) and San Luis Obispo Unit (SLU) even became the new hot spots. Meanwhile, the coverage area of the original human-caused wildfire hot spots on the south coast has been further expanded. From 1920 to 1999, the density of human-caused wildfires in the Sierra Nevada Mountain was very low in central California. Still, in the past two decades, it has become a new wildfire ignition hot spot. The counties in northern California, such as Siskiyou Unit (SKU), Shasta-Trinity Unit (SHU), Tehama-Glenn Unit (TGU), etc., have been almost no human-caused wildfires from 1920 to 1999, but widespread human-caused wildfires have emerged in the past two decades.

After inducing the wildfire burned area into the KDE calculation, the spatial density distribution has changed significantly. In general, as shown in 7a,d, the regions where large wildfires are concentrated are SKU and Sonoma-Lake-Napa Unit (LNU) in Northern California and MVU in the South Coast. Although the number of wildfires in the central Sierra Nevada Mountains has increased significantly, the total burned area did not significantly change. Thereafter, the wildfires with different causes were separated, and it can be found from 7b,e that natural wildfires with large burned areas were concentrated in northern California. In the past two decades, the region with a very high-density of wildfire occurrence in the northernmost SKU has expanded significantly, and a new hot spot of wildfires has also appeared in Lassen-Modoc Unit (LMU). However, the high-density wildfire area between Tuolumne-Calaveras Unit (TCU) and Madera-Mariposa-Merced Unit (MMU) did not arise in the past two decades. In the distribution of human-caused wildfires, as shown in 7c,f, the density of wildfires in MVU in the southernmost part of California has surpassed that of historical hot spots, VNC and LAC. Meanwhile, the density of wildfires at the junction of TCU and MMU in the central region has also increased.

Comparing 6 and 7, it is obvious that the spatial distribution of wildfire density and burned area-weighted wildfire density are not entirely consistent. CAL FIRE Units along the South Coast, which are in the climate division of South Coast Drainage, are prominent in both densities, and are mainly composed of human-caused wildfires. The SKU and LMU units in the northernmost part of North Coast Drainage are the areas where natural wildfires were concentrated, and the distribution of SKU wildfires is relatively wider. The Units adjacent to the Sierra Nevada Mountains in central California, which are the units in the northeast of San Joaquin Drainage, show a low wildfire density when the burned area was added to the calculation, even though the number of wildfires has increased rapidly in the past two decades. This distribution is related to the vegetation cover and land use in California. In northern California, the evergreen and deciduous forests are the dominant vegetation, the forests are dense and less developed by human, and the population density is relatively low^28,29^. Wildfires are difficult to be detected early-on in these remote areas, and there is enough fuel to keep them burning and spreading. On the other hand, shrubs are the dominant vegetation in southern California. Also, most of the southern CA areas have been developed and associated with a higher level of human activity, leading to wildfires in southern California has a greater social and economic impact on human lives and society^30^.

**Figure 8:**
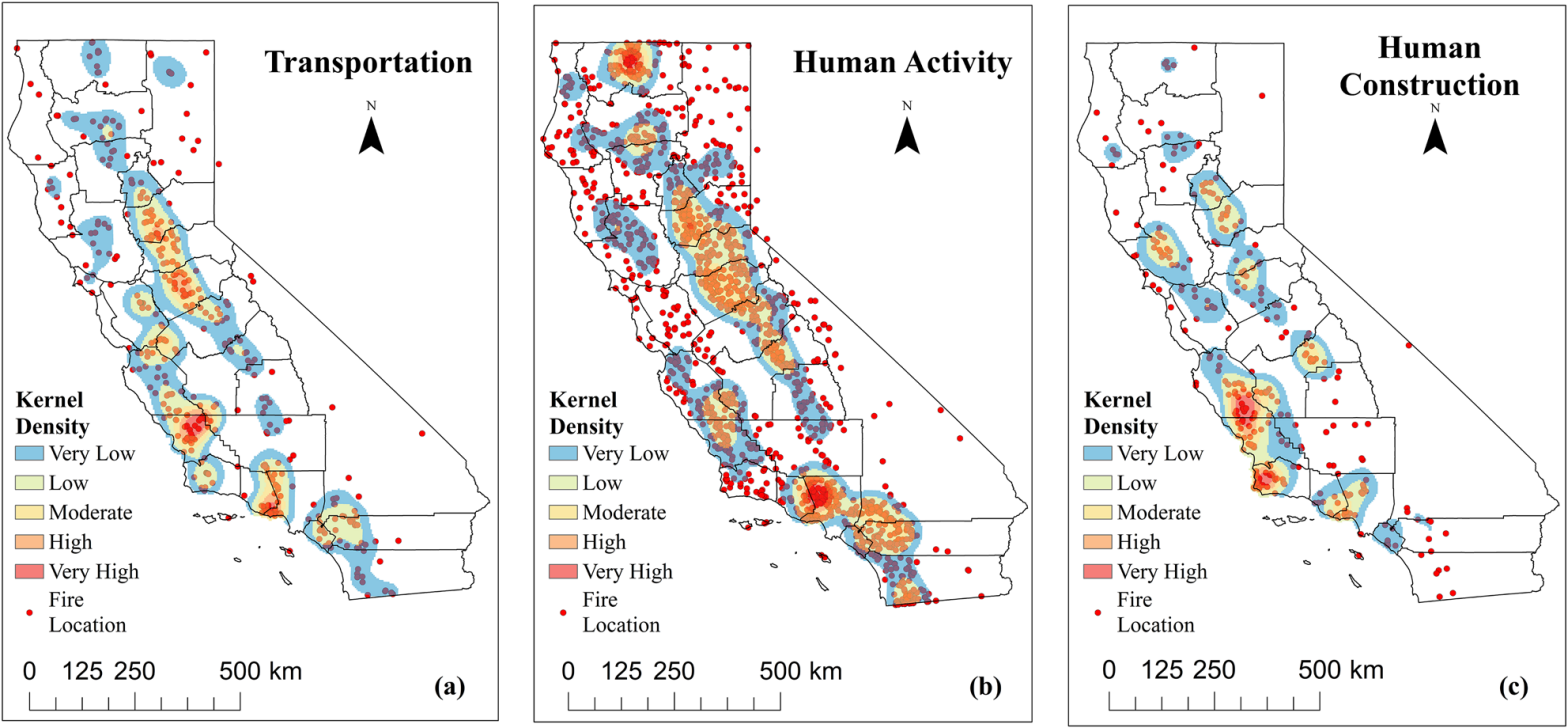
KDE Analysis of human-caused wildfires in CA from 2000 to 2019. (**a**) Transportation (railroad, vehicle, aircraft); (**b**) Human Activity (equipment use, smoking, campfire, debris, arson, playing with fire, firefighter training, non-firefighter training, escaped prescribed fire, illegal alien campfire); (**c**) Human Construction (power line, structure).

From the discussion above, it can be found that while the frequency and spatial density distribution of human-caused wildfires have changed significantly in the past two decades, the changes in burned area were relatively small because of the high proportion of small wildfires. Also, unlike natural fires, human-caused fires can be prevented or controlled in the early stage by taking effective measures^19^. Therefore, the human-caused wildfires were further classified to generate a more detailed spatial density distribution map. The anthropogenic causes were subdivided by CAL FIRE into 15 types. The spatial distribution of wildfires with different causes are shown in the supplementary figures (Supplementary Fig. 1). In this study, human-caused wildfires were classified into three categories: transportation (railroad, vehicle, aircraft), human activity (equipment use, smoking, campfire, debris, arson, playing with fire, firefighter training, non-firefighter training, escaped prescribed fire, illegal alien campfire) and construction (powerline, structure). As shown in Fig. 8, hot spots for all three broad types of wildfires include areas along the Sierra Nevada Range and along the southern coast. However they differ in the density level and coverage. Among them, the number and coverage of wildfires caused by human subjective behavior are larger than those caused by traffic and construction. Besides, the wildfires caused by human activities also led to the emergence of a unique hot spot in the northernmost edge of CA, which is the SKU county. Therefore, for the wildfire management purpose, it would be proactive to provide wildfire education to residents in regions with high wildfire risk, update the wildfire risk map in time, and issue early warnings of wildfire risk to the public during the fire season, to increase the public’s awareness of wildfire prevention.

### Multivariate analysis of California wildfires

The occurrence and spread of wildfires are related to human activities and environmental variables. In order to formulate effective suppression and control policies for wildfire management, it is essential to understand the relationship between the spatial distribution of wildfires and various variables. From the KDE analysis, the spatial distributions of the wildfire density calculated with and without burned area were obtained, which also shows the areas with high wildfire risk from 2000 to 2019. According to the research from Faivre et al.^7^, 12 variables that have potential correlations with wildfires, involving human-related variables, geographic conditions, fuel, and climate variables were selected to conduct the subsequent analyses.

Table 4 calculated the spatial correlation between the burned area-weighted wildfire density and potential anthropogenic and environmental variables within the wildfire perimeters, as well as the interrelation between each variable. It can be derived from the first column that among the human-related variables, except for the distance to the road, other variables are positively correlated with the wildfire occurrence density. It means that in areas where wildfires have occurred in the last two decades, the farther away from the power line, the higher the wildfire density; the closer to the road, the higher the wildfire density; and the greater the density of houses and population, the higher the density of wildfires. Among environmental variables such as topography, vegetation cover, and climate, only elevation is negatively correlated with wildfire density. That is, the higher the elevation, the lower the wildfire density. From the correlations among various variables, it can be found that there is a strong correlation between the distance from the wildfire perimeter to the road and power line, population, and house density, as well as elevation and two climate variables. For further analyses, one variable would be removed between the two variables whose correlation is greater than 0.5. Therefore, the distance to power line, population density and elevation were removed in the multivariate analysis.

**Table 4:** Spatial Correlation Analysis between 12 selected variables wildfire occurrence density: distance to power line (DP), distance to road (DR), housing density (DH), population density (DP), elevation, aspect, slope, tree, shrub, grass, maximum temperature (Tmax), maximum vapor pressure deficit (VPDmax).

	Wildfire occurrence density	vpd max	t max	Tree	Shrub	Herb	Slope	Elevation	Aspect	Population density	Housing density	Distance to road
DP (km)	0.0153	−0.3949	−0.4819	0.0116	−0.0371	−0.0065	0.1136	0.4290	−0.0067	−0.1620	−0.1626	0.5524
DR (km)	−0.0083	−0.3112	−0.3867	0.0193	−0.0367	−0.0028	0.0039	0.2895	−0.0216	−0.1609	−0.1617	
DH (houses/km$^2$)	0.0204	0.1268	0.2019	−0.0086	−0.0162	0.0016	−0.0904	−0.1880	0.0042	0.8132		
DP (persons/km$^2$)	0.0179	0.1250	0.2059	−0.0085	−0.0189	0.0027	−0.0732	−0.1908	0.0051			
Aspect ($^{\circ }$)	0.0203	−0.0315	−0.0052	0.0470	0.0291	0.0079	0.0832	0.0018				
Elevation (km)	−0.0375	−0.6086	−0.8359	−0.0771	−0.0398	0.0035	0.0017					
Slope ($^{\circ }$)	0.0778	−0.0797	−0.0159	0.1342	0.0579	0.0048						
Grass ($\%$)	0.0193	0.0176	0.0207	0.1331	0.0212							
Shrub ($\%$)	0.0503	−0.0174	0.0258	0.0570								
Tree ($\%$)	0.0316	0.0598	0.1085									
Tmax ($^{\circ }C$)	0.0734	0.0501										
VPDmax (hPa)	0.0501											

**Figure 9:**
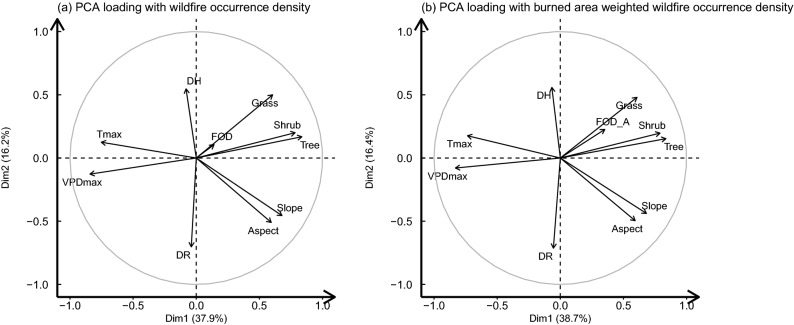
PCA loading plots with (**a**) fire occurrence density, (**b**) burned area weighted fire occurrence density. The variables include distance to road (DR), housing density (DH), aspect, slope, tree, shrub, grass, maximum temperature (Tmax), maximum vapor pressure deficit (VPDmax), wildfire density (FOD) and burned area weighted wildfire density ($FOD_A$).

The principal component analysis (PCA) was implemented on the remaining variables and the two types of wildfire spatial densities obtained from KDE, to classify the variables and evaluate their relationships. The eigenvalue matrix was attached in the supplement information (Supplementary Table S3.). Both PCA results require five principal components to explain at least 80$\%$ of the data variance. The interrelations of the variables and the fire occurrence density decomposed by PC1 and PC2 are shown in Fig. 9. There is a strong and similar interrelationship between the two types of fire densities and the driver variables. The length and orientation of the variables indicate that the wildfire densities have the strongest correlation with the grass cover and the other two variables of vegetation cover (shrub and tree), namely fuel cover in general. Meanwhile, the correlation between the climate variables and the wildfire densities is also significant, especially for the maximum vapor pressure deficit (VPDmax). Besides, the human-related variables are moderately correlated with the wildfire densities, while topographic variables are almost orthogonal with the wildfire densities, which means their correlations are weak.

Based on the analyses above, the Logistic Regression (LR) was implemented on the selected nine variables to further determine their relationship with wildfire occurrence. The coefficient, standard error and the significance level for each variable were shown in Table 5. The positive and negative sign of the coefficient represents the positive or negative correlation with the wildfire occurrence, and the p-value indicates whether the correlation is significant. The results reveal that the climate variables are the most critical in whether the wildfires can be ignited or not, followed by the variables of distance to road, and the cover of grass. The sign of the coefficient of the human-related variables is negative, which means that in general, wildfires ignited far from the human communities. Similarly, the areas where trees are dominant vegetation cover have fewer wildfire ignitions. Overall, logistic regression results show that the areas with high temperature, high VPD, grass as the dominant vegetation cover, and away from human communities have a higher risk of wildfire ignition.

**Table 5:** Logistic regression results of uncorrelated explanatory variables for California wildfires occurrence (2000–2019).

Variables	Logistic regression		
	Coefficient	S.E.	p-value
Distance to roads (km)	−0.0354	0.0021	$<0.0001$
Housing density (houses/km^2^)	−0.0017	0.0003	$<0.0001$
Slope ($^{\circ }$)	0.0059	0.0003	$<0.0001$
Aspect ($^{\circ }$)	0.0143	0.0024	$<0.0001$
Tree ($\%$)	−0.0070	0.0011	$<0.0001$
Shrub ($\%$)	0.0009	0.0013	0.5000
Grass ($\%$)	0.0230	0.0011	$<0.0001$
Max temperature ($^{\circ }C$)	0.0934	0.0081	$<0.0001$
Max vapor pressure deficit (hPa)	0.0655	0.0058	$<0.0001$

## Conclusion

This study investigated the temporal and spatial distribution of wildfires with different sizes and causes from 2000 to 2019 in California. The wildfires between 1920–1999 were added into the analyses for comparison. Also, the relationships among the explanatory anthropogenic and environmental variables and wildfires were analyzed by principal component analysis and Logistic regression.

Our study found that the distribution of wildfire size after the year 2000 fits the truncated Pareto well, while using generalized Pareto to describe the fire size distribution would be more appropriate if the 80 years of historical wildfires from 1920 to 1999 are also taken into account. After taking the logarithm of the wildfire burned area for fitting, we found that the shape of the empirical probability density histogram of human-caused wildfires changed greatly, which is mainly reflected in the significant increase in the number of small fires with an area of less than 100 acre in the past 20 years. It directly leads to the change in the probability density distribution of all wildfires.

Comparing the temporal distributions of wildfires in the past two decades, and the earlier 80 years (1920–1999), we found that the frequency and total burned area of all wildfires have increased significantly. The start time and peak months of the wildfire season have been advanced, and the covered months have been lengthened. For large and small wildfires, the annual frequency of large wildfires has remained stable for the last 100 years, but the total burned area has increased rapidly in the past two decades, along with the obvious increase in the uncertainty. It illustrates that the comprehensive environmental conditions, such as changes in climate and vegetation, have increased the coverage of potential wildfire ignitions. On the other hand, for the small wildfires, although the growth rate of frequency has increased significantly, the total burned area has remained stable. Among the wildfires of different causes, the frequency of human-caused wildfires has increased the most, which shows that for contemporary wildfire management, enhancing public awareness of wildfire prevention is also of importance. The trends in the seasonal distribution of different types of wildfires are relatively consistent.

In the spatial distribution, the spatial density distribution of wildfires without being weighted by burned area has changed significantly in both time periods (1920–1999 and 2000–2019). The hot spots for natural and human-caused wildfires have grown outwards in the last two decades compared to the 1920–1999 hot spot areas. Human-caused wildfires have even emerged new hot spots, which are along the west coast and the Sierra Nevada mountain range, and the variability in their spatial distribution has also greatly increased. In the spatial distribution of burned area-weighted wildfire density, natural wildfires became more concentrated in Northern California. The original hot spots at the junction of TCU and MMU have lessened in the past two decades. In terms of human-caused wildfires, their distribution in central California became more concentrated, while the distribution in the southern part tends to be scattered. After taking the burned area into account, the uncertainty of the spatial distribution of the total area of human-caused wildfires is greatly reduced.

In terms of the causes of wildfire occurrence and growth, the spatial correlation analysis and principal component analysis reveals the interrelation between the selected variables. Apart from elevation and the distance from the historical wildfires to roads, which are negatively correlated with the density of wildfire occurrence, all the other variables have positive correlations with it. Among them, slope, temperature and maximum vapor pressure deficit have positive correlation with wildfire occurrence. It can be derived that natural factors, especially climate variables, have a greater impact on the density of wildfires in regions where wildfires have occurred. The subsequent PCA analysis expanded the study region to the entire state of California, analyzing the relationship between these variables and whether or not a wildfire has ever occurred. The results show that the vegetation cover and climate have a significant contribution to the occurrence of wildfires, especially the percentage of grass cover and the maximum vapor pressure deficit. Furthermore, we find out that California’s wildfires tend to be ignited in the region with high temperature, high vapor pressure deficit, wide grass cover, and away from the human community.

## Methods

### Study area

**Figure 10:**
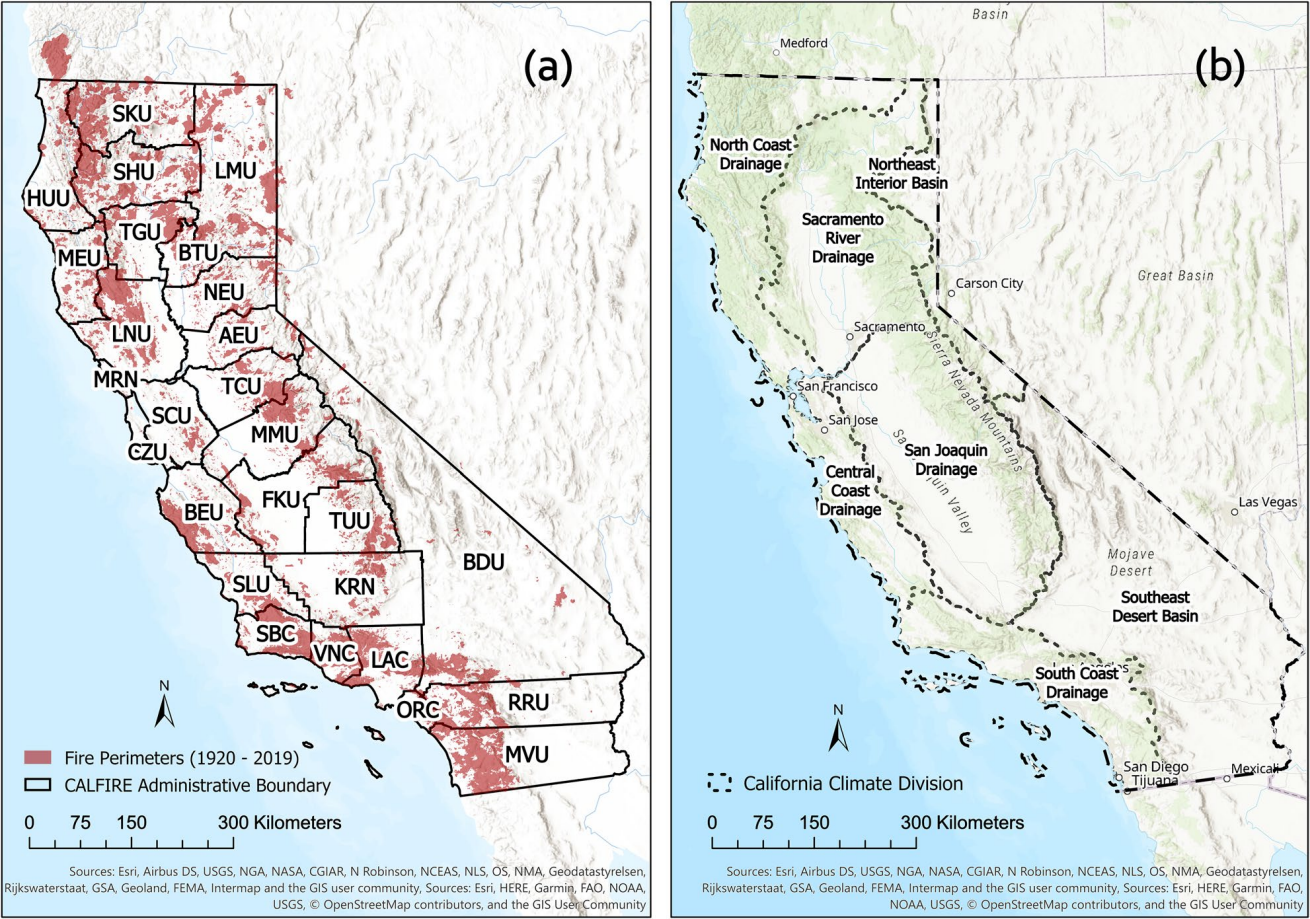
Study region division in California: (**a**) California Department of Forestry and Fire Protection (CAL FIRE) Administrative Units; (**b**) California climate divisions from National Oceanic and Atmospheric Administration (NOAA).

California (CA) is located in the western United States and has a Mediterranean climate. Summers in CA are hot and dry, and rainfall is concentrated in winter. The vegetation coverage in CA is about one-third of the total area, and according to the United States National Land Cover Database (NLCD), the main vegetation types are shrubs, evergreen forest and herbaceous (39.03$\%$, 18.59$\%$, and 13.47$\%$)^31^. In addition, over 147 million trees have died since 2010 across the state^32^. Dead vegetation accumulated in forests could be easily ignited by lightning, thunderstorms or sparks left by human activities. Moreover, each year from September to May, the dry Santa Ana wind, with high desiccating potential and high wind speed, arrives from the Great Basin and the Mojave Desert in the southwestern inland crossing the mountains. This addition of strong wind forces means that even small ignition sources have the risk of developing into extreme wildfires. The natural environmental conditions of CA make it a high-risk area for wildfires. To make the analyses and conclusions more practical, wildfires were analyzed mainly by the California Department of Forestry and Fire Protection (CAL FIRE) Administrative Units. The climate divisions were also added to summarize the wildfire spatial distribution characteristics. The maps of these two regional divisions are shown in Fig. 10.

### Data

The historical wildfire records in CA along with start time, burned area, fire perimeter and the causes of ignition were extracted from the CAL FIRE database (https://frap.fire.ca.gov/mapping/gis-data), which contains statewide wildfire records under the protection of multiple agencies. Their latest fire dataset was updated in May 2020. There is a minimum burned area requirement for wildfires to be included in this database, which is 10 acres for timber fires, 30 acres for brush fires, and 300 acres for grass fires. This study focused on the wildfire events from 2000 to 2019, meanwhile, wildfires from 1920 to 1999 were also selected to compare with recent wildfires in terms of frequency, burned area and ignition causes, so as to analyze the characteristics of recent wildfires. The sample size of wildfire records covered in this study is 17193, of which 5234 were from 2000 to 2019.

To explore the relationships among different environmental conditions, human activities and wildfires, a series of explanatory variables from 2000 to 2019 were selected, and explored via statistical analyses. In terms of the environmental conditions, two to three representative variables were selected from each aspect in the wildfire behavior triangle (weather, fuels, and topography)^33^. The human-related variables were selected according to Faivre et al.’s^7^ and Ruffault and Mouillot’s^34^ research. The list of variables, their released time and covered time range and their sources were shown in Table 6.

### Statistical analysis

Several statistical methods were used to obtain the distribution of wildfires and the relationship between environmental variables and wildfire occurrences.

Wildfires, as common extreme climate events in California, has an obvious heavy-tailed feature in their frequency distribution^16^, which means that the majority of wildfires (99$\%$) are small, while the remaining 1$\%$ of large wildfires would be responsible for the majority of the damage. Several studies have indicated that the Generalized Pareto or truncated Pareto distribution can depict the wildfire size distribution very well^17,35^. To evaluate the wildfire size distribution in entire California, some typical distributions, including gamma, exponential, Weibull, Generalized Pareto and truncated Pareto distribution were selected for fitting. The goodness of fit were measured comprehensively by Akaike information criterion (AIC), Kolmogorov-Smirnov (KS) test and Cramer-Von Mises (CvM) Test^36^. To better understand the differences in the spatial and temporal distribution of wildfires with different sizes, wildfires were divided into large and small two groups. The threshold of the large wildfires was decided from the mean excess plot. In the mean excess plot, when the threshold and the mean excess over this threshold display a linear relationship, the exceedance over these threshold fits the Generalized Pareto distribution^17,24^.

Within the temporal analysis, the wildfire frequency and burned area were plotted with year. Then the segmented linear regression was implemented on these plots to show the trend of wildfires^37^. The coefficient of determination ($R^2$) and the p-value (p) were added to the plots to indicate the goodness of fit of the regression equation.

In the spatial analysis, Kernel density estimation (KDE) was implemented on the fire occurrence points in ArcGIS to identify the hot spots of wildfires, where indicated the region with high wildfire occurrence density. KDE calculated the density of ignition points in a neighborhood around those points, and assigned the density values to cells to make up an intensity map. Conceptually, a smoothly curved surface is fitted over each point. The surface value is highest at the location of the point and diminishes with increasing distance from the point, reaching zero at the search radius distance from the point^38^. The equation of KDE at a location is shown below:

1 $$\begin{aligned} f(x)=\frac{1}{R^2} \sum ^n_{i=1} \lbrace \frac{3}{\pi }P_i\left( 1-\left( \frac{d_i}{R}\right) ^2\right) ^2 \rbrace \end{aligned}$$

where f(x) represents the density, R is the search radius, $p_i$ is population field of the point i, $d_i$ is the distance between the point i and the location^38^. The population field is used to adjust the weight of each point. In this study, the population field was set to 1 and burned area separately, to show the wildfire occurrence density and burned area-weighted wildfire density. The kernel density estimation makes full use of the input data itself and avoids the subjective introduction of prior knowledge, so as to achieve the maximum approximation of the sample data.

The variation of the spatial and temporal distribution of wildfires is related to a lot of variables. In this study, twelve representative explanatory variables were selected to be further analyzed. To get the relationship between these variables and the occurrence of wildfires, the spatial correlation analysis was implemented to filter out the redundant variables. The correlation between the layer of variable and the layer of wildfire density which was obtained from KDE were calculated as below:

2 $$\begin{aligned} Corr_{ij}&= \frac{Cov_{ij}}{\delta _i \delta _j} \end{aligned}$$

3 $$\begin{aligned} Cov_{ij}&= \frac{\sum ^{N}_{k=1}(Z_{ik} - \mu _i)\left( Z_jk - \mu _j\right) }{N-1} \end{aligned}$$

where Z is the cell value in each layer, i and j represent the layers, $\mu$ is the mean of a layer, N is the total number of cells and K represents a specific cell^39^.

To find out the interrelationship between the variables and whether there really is a relationship between the variables and wildfire density, the Principal Component Analysis (PCA) was performed. PCA is a method for preprocessing high-dimensional data. It can find the most important features, remove noise and unimportant features, and reduce the dimensionality by orthogonal decomposition of the original variables. The general process of PCA is as follows: decentralize the samples, calculate the covariance matrix of each variable, figure out the eigenvalues of the covariance matrix and the corresponding eigenvectors, sort the eigenvectors according to the eigenvalues, and interpret the principal components according to the eigenvectors. The absolute value of the corresponding coefficient of the original variable in each principal component represents the importance of the variable in this component^40^.

To obtain the relationship between these variables and fire occurrence, that is the presence or absence of ignitions in each administrative unit, the logistic regression was implemented referring to the study of Faivre et al.^7^. As a generalized linear regression model, logistic regression can dichotomize the dependent variables through the attributes of multiple independent variables^41^. The equation of the logistic regression is shown in equation (1):

4 $$\begin{aligned} \ln \left( \frac{P}{1-P}\right) = w_0 + w_1x_1 + \cdots + w_n x_n \end{aligned}$$

where P represents the probability of the wildfire occurrence, x represents various characteristics of the samples and w represents the weight of the x. To correlate the occurrence of the wildfires with the explanatory variables, California was divided into 3 km $\times$ 3 km cells, with a total number of 73,455 cells. 5,177 cells among them were marked as having been on fire during the past 20 years. The variable data in each cell were averaged and integrated. After the training of the logistic regression, the weight of each variable in the determination process, that is, the influence of various natural or human factors on the occurrence of wildfires, was obtained.

**Table 6:** List of wildfire-related variables.

Variables	Source
Distance to major roads	Census Bureau
Distace to powerline	California Energy Commission
Population density	Census Bureau
Housing density	Microsoft Building Footprint
Elevation	USGS (National Elevation Database)
Slope	USGS (National Elevation Database)
Aspect	USGS (National Elevation Database)
Tree	LANDFIRE (Fuel Vegetation cover)
Shrub	LANDFIRE (Fuel Vegetation cover)
Herb	LANDFIRE (Fuel Vegetation cover)
Max Temperature	PRISM
Max vapor pressure deficit	PRISM

## Supplementary information

Supplementary Information.

## Data Availability

The datasets generated during and/or analysed during the current study are available from the corresponding author on reasonable request.
