# Supplementary material for: Spatial and temporal pattern of wildfires in California from 2000 to 2019
Source: Sci Rep. 2021 Apr 22;11:8779. doi: 10.1038/s41598-021-88131-9 (PMC8062671; doi:10.1038/s41598-021-88131-9)
Supplement: Supplementary file 1 — Supplementary Information. [file 41598_2021_88131_MOESM1_ESM.pdf]

Spatial and temporal pattern of wildfires in California from 2000 to 2019

Shu Li[1,*] and Tirtha Banerjee[1]

[1]Department of Civil and Environmental Engineering, University of California, Irvine, Irvine, CA 92697, USA

[*]To whom correspondence should be addressed. E-mail: shul15@uci.edu

Supplementary Tables

		Estimated Parameter		Goodness of fit test results		
		Shape (α)	Scale ($beta$)	AIC	KS	CvM
All wildfires (1920-1999)	Lognormal	0.848	0.322	26,705.3	0.073	18.945
	Weibull	3.730	2.720	25,836.4	0.024	1.072
	Gamma	11.330	4.620	26,172.3	0.053	9.850
Natural wildfires (1920-1999)	Lognormal	0.704	0.379	3,309.8	0.052	1.148
	Weibull	2.870	2.440	3,418.4	0.075	1.731
	Gamma	7.130	3.290	3,323.9	0.053	1.049
Human-caused wildfires (1920-1999)	Lognormal	0.776	0.352	4,158.6	0.059	1.957
	Weibull	3.270	2.570	4,143.3	0.044	0.546
	Gamma	8.990	3.900	4,115.8	0.044	1.219
All wildfires (2000-2019)	Lognormal	0.663	0.375	11,209.8	0.048	3.391
	Weibull	2.800	2.340	11,844.6	0.088	8.936
	Gamma	6.860	3.300	11,358.5	0.057	3.641
Natural wildfires (2000-2019)	Lognormal	0.743	0.401	3,189.5	0.065	1.598
	Weibull	2.770	2.560	3,254.3	0.072	1.999
	Gamma	6.530	2.870	3,195.0	0.070	1.631
Human-caused wildfires (2000-2019)	Lognormal	0.642	0.358	4,345.2	0.048	1.100
	Weibull	2.920	2.280	4,609.3	0.088	3.097
	Gamma	7.540	3.720	4,399.0	0.055	1.097

Table 1: The fitting parameters of frequency distribution of the logarithm of wildfire burned area and the goodness of fit test results

Fire Frequency		slope	intercept	Burned Area		slope	intercept
All wildfires	1920-1999	1.31	-2,434	All wildfires	1920-1999	0.84	-1,392
	2000-2019	5.32	-10,445		2000-2019	21.70	-42,921
Large wildfire	1920-1999	0.24	-426	Large wildfire	1920-1999	0.74	-1,215
	2000-2019	-1.29	2,650		2000-2019	21.64	-42,834
Small wildfires	1920-1999	1.11	-2,077	Small wildfires	1920-1999	0.10	-179
	2000-2019	5.87	-11,614		2000-2019	-0.22	462
Naturalwildfires	1920-1999	0.47	-904	Naturalwildfires	1920-1999	0.70	-3,543
	2000-2019	2.02	-3,990		2000-2019	6.18	-19,123
Human-caused wildfires	1920-1999	1.05	-2,033	Human-caused wildfires	1920-1999	2.09	-4,042
	2000-2019	2.74	-5,404		2000-2019	7.18	-14,233

Table 2: Segmented linear regression coefficients for temporal distribution of wildfire frequency and burned area

Variable	PC Coefficients					PC Coefficients				
	PC1	PC2	PC3	PC4	PC5	PC1	PC2	PC3	PC4	PC5
Distance to Roads (km)	-0.020	-0.552	0.243	-0.259	0.058	-0.028	-0.557	0.169	-0.274	0.353
Housing Density (houses/km2)	-0.042	0.429	0.211	-0.290	0.818	-0.034	0.436	0.062	-0.802	0.355
Aspect (°)	0.305	-0.400	-0.246	0.090	0.375	0.301	-0.387	-0.309	-0.145	0.167
Slope (°)	0.348	-0.358	-0.204	0.146	0.296	0.347	-0.342	-0.297	-0.091	0.095
Tree (%)	0.429	0.132	-0.057	0.170	0.028	0.425	0.119	-0.022	0.121	0.171
Shrub (%)	0.402	0.157	-0.135	0.231	-0.003	0.401	0.154	-0.115	0.181	0.259
Grass (%)	0.311	0.393	-0.093	0.161	-0.159	0.309	0.375	0.032	0.279	0.354
Max Temperature ($^\circ C$)	-0.386	0.098	-0.423	0.355	0.199	-0.374	0.137	-0.477	0.161	0.393
Max Vapor Pressure Deficit (hPa)	-0.432	-0.100	-0.354	0.284	0.134	-0.422	-0.062	-0.422	0.138	0.253
Fire Occurrence Density	0.072	0.083	-0.679	-0.710	-0.124	-	-	-	-	-
Fire Occurrence Density with Burned Area	-	-	-	-	-	0.179	0.177	-0.603	-0.287	-0.522
Cumulative proportion	0.379	0.541	0.649	0.739	0.823	0.387	0.551	0.666	0.756	0.824

Table 3: Principal component coefficients for wildfire-related variables and wildfire density with and without burned areas weighting.

Supplementary Figures

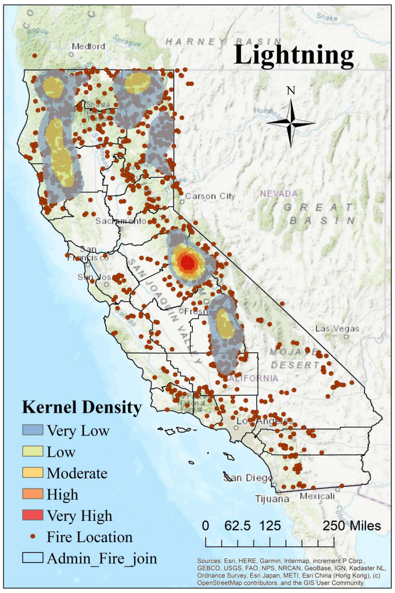

(a) lightning

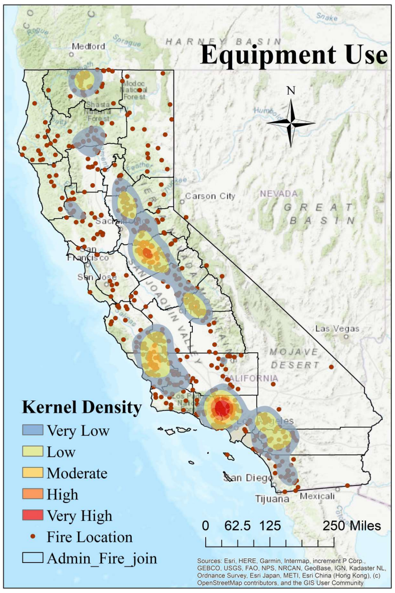

(b) equipment use

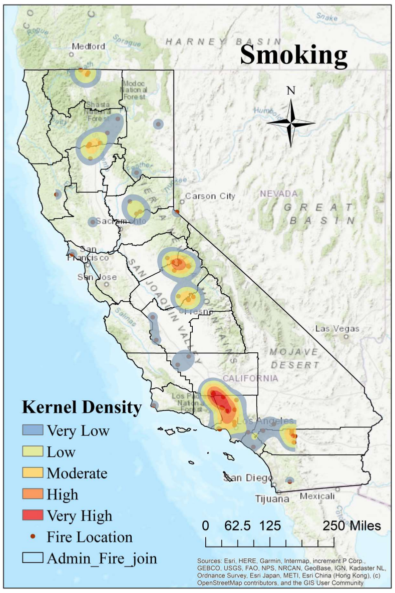

(c) smoking

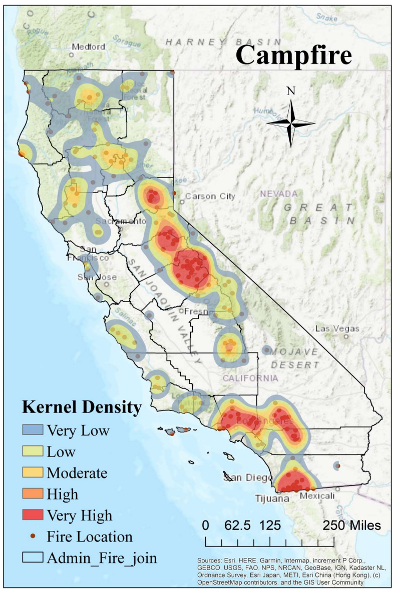

(d) campfire

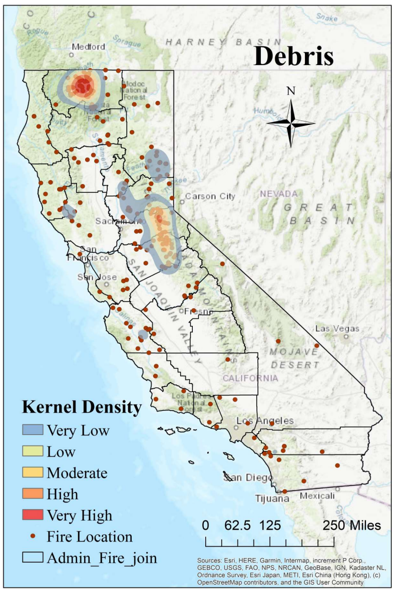

(e) debris

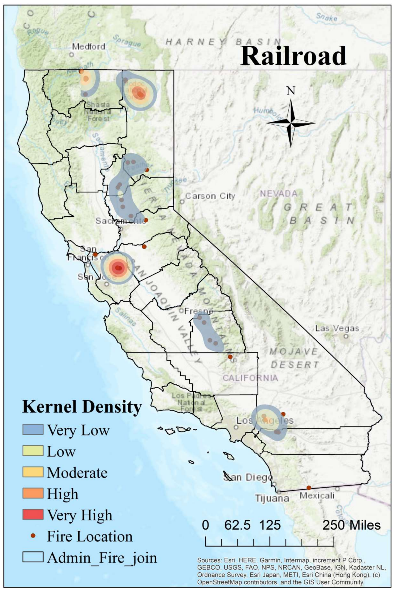

(f) railroad

Supplementary Figure 1: KDE results for wildfires with different causes

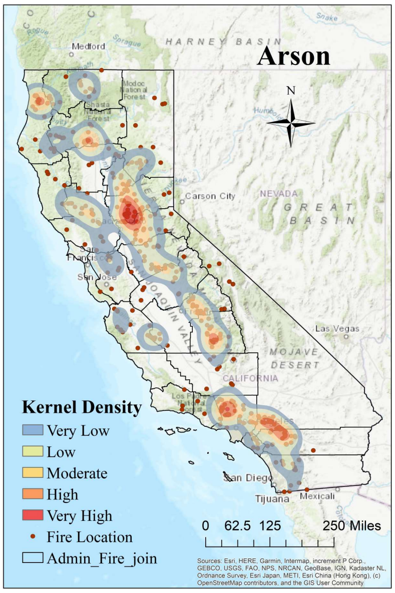

(g) arson

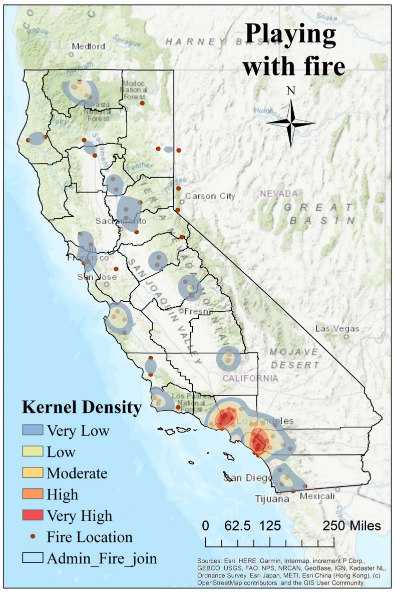

(h) playing with fire

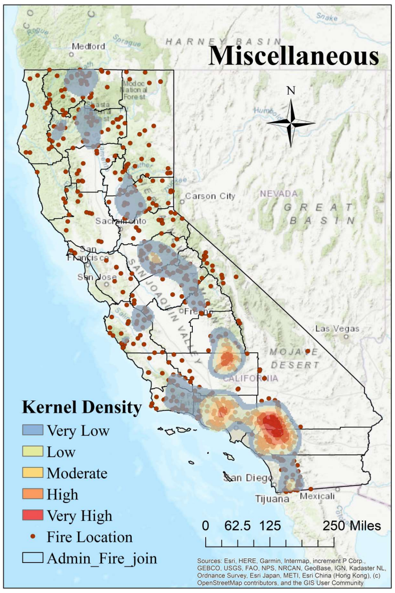

(i) miscellaneous

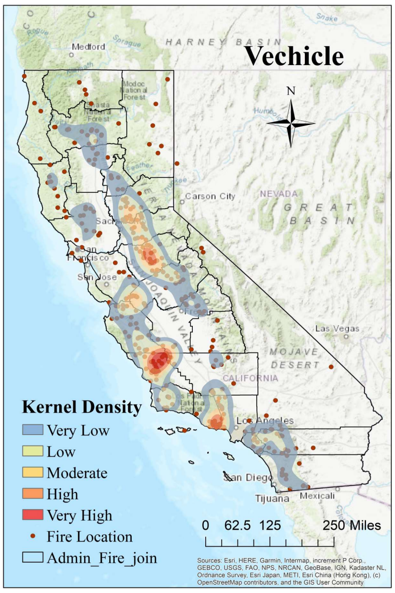

(j) vehicle

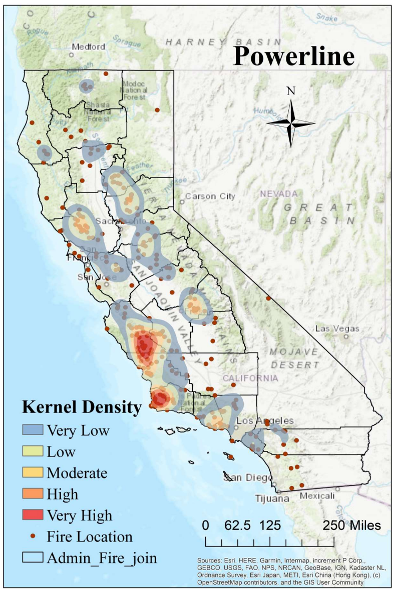

(k) power line

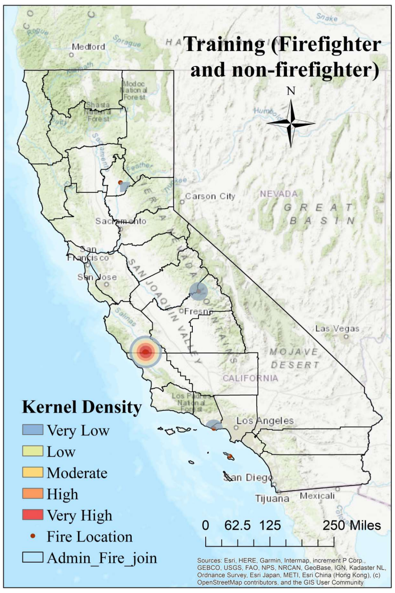

(l) training

Supplementary Figure 1: KDE results for wildfires with different causes

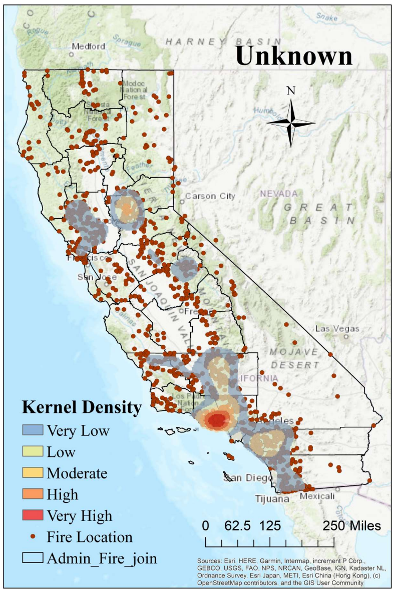

(m) unknown

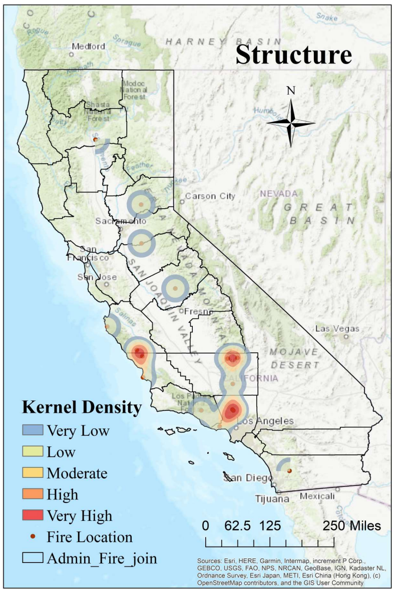

(n) structure

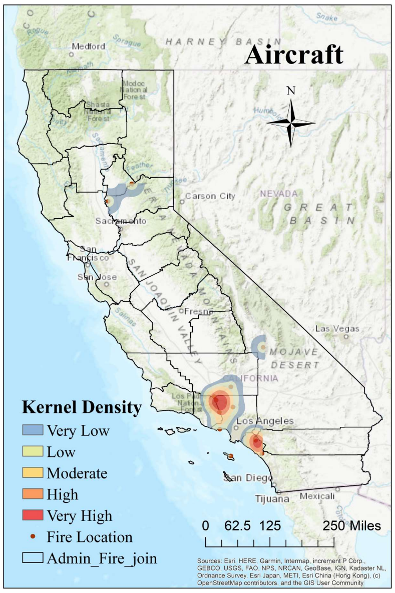

(o) aircraft

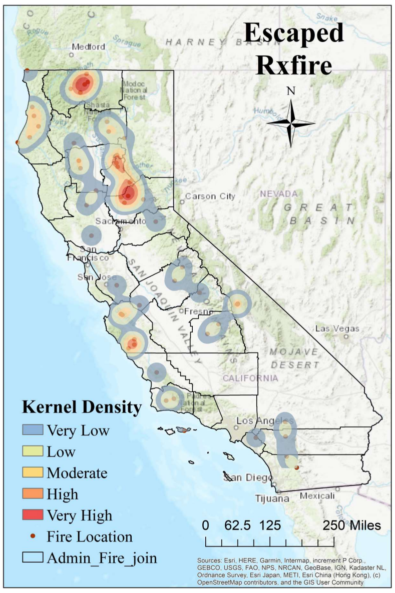

(p) escaped Rxfire

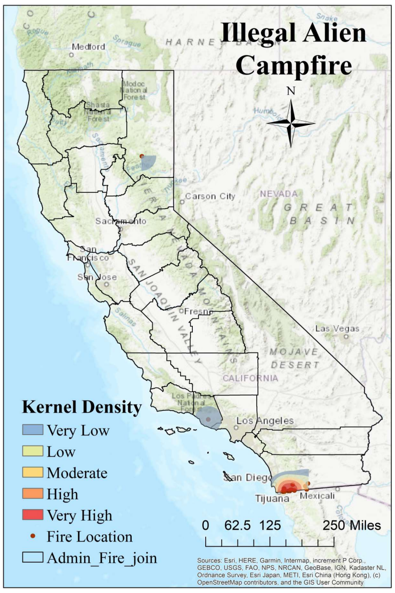

(q) illegal alien campfire

Supplementary Figure 1: KDE results for wildfires with different causes
